# Advanced 3D Horse Reconstruction: Integrating Image-to-3D Modelling and Non-Rigid Registration for On-Barn Body Measurements

**DOI:** 10.3390/ani16152397

**Published:** 2026-08-03

**Authors:** Xufeng Yuan, Shubin Wang, Qitao Zhu, Yiwei Wang, Alexey Ruchay, Andrea Pezzuolo, Qin Wang, Jiangong Li, Hao Guo

**Affiliations:** 1 College of Land Science and Technology, China Agricultural University, Beijing 100083, China; 2 College of Information and Electrical Engineering, China Agricultural University, Beijing 100083, China; 3 Inner Mongolia Shengmu High Tech Dairy Co., Ltd., Dengkou Industrial Park, Dengkou County, Bayannur City 015200, China; 4 Faculty of Economics, Peoples’ Friendship University of Russia, Moscow 117198, Russia; 5 Department of Land, Environment, Agriculture and Forestry, University of Padova, 35020 Legnaro, Italy; 6 College of Animal Science and Technology, China Agricultural University, Beijing 100193, China

**Keywords:** precision livestock farming, animal housing, three-dimensional reconstruction, computer vision, artificial intelligence

## Abstract

Precision livestock farming (PLF) relies on high-precision three-dimensional (3D) horse reconstruction and automatic body measurement to support refined breeding management and health surveillance. However, data collection is restricted by building environment noise and hardware layout constraints; complex equine body shapes and large individual variations induce local geometric distortions in reconstructed models, limiting field deployment. Drawing on generative 3D reconstruction, this study develops the first image-to-3D pipeline that leverages three consumer depth cameras to reconstruct high-fidelity 3D horse models, integrating reconstruction, non-rigid optimisation and automatic body measurement. The image-to-3D module accurately extracts core morphological traits such as torso outlines and limb ratios for initial reconstruction. To eliminate local geometric deformation and recover scene scale, coarse-to-fine non-rigid fitting optimisation with dynamic surface feature matching weights is proposed, strengthening alignment between reconstructed meshes and real horse anatomical structures. Comparative experiments on multiple equine datasets verify that our method surpasses existing algorithms in measurement precision and reconstruction integrity. Compared with baseline methods, the non-rigid registration reduces core body measurement errors and Chamfer Distance (CD) by over 50%, while increasing the F-Score by more than 20%. This work enables automatic horse body phenotyping and offers technical references for image-to-3D dimensional measurement of other livestock species in large-scale precise breeding.

## Simple Summary

Precise body measurement of horses is the core foundation of equine breeding evaluation, daily health monitoring and standardised horse farm management. Traditional manual measurement with tape measures and callipers is labour-intensive and prone to human error and causes severe stress to horses during capture and restraint. Current camera-based three-dimensional (3D) reconstruction techniques can reconstruct horse body models automatically, yet most existing solutions generate distorted limb and torso meshes and fail to adapt to complex real breeding barn environments with fences, feed piles and light interference. This work proposes a low-cost three-camera measurement system to reconstruct high-fidelity 3D horse models. The integrated non-rigid registration strategy automatically corrects local geometric distortion and realises fast, stable calculation of core body indicators including wither height, body length, heart girth and cannon circumference. Comparative experiments on two independent horse datasets demonstrate that our method reduces measurement errors by over 50% and improves reconstruction integrity significantly relative to mainstream existing technologies. This low-cost, easily deployed system lowers the technical threshold of automated equine phenotyping, facilitates large-scale standardised horse breeding and health tracking, and has the potential to provide transferable technical references for body measurement of cattle, sheep and other livestock species.

## 1. Introduction

The accurate acquisition of livestock morphological features and body dimension parameters is a key basis in breeding improvement, health monitoring, production performance evaluation, and precision agricultural management. Livestock body measurements serve as an essential bridge for studying the relationships between individual animal characteristics and their genetic and environmental factors [1]. It covers the manifestations of animals across multiple aspects, such as morphology, structure, and function, provides abundant biological information, and is widely applied in fields including genetics, reproduction, and animal health management [2].

In horse body measurement, phenotypic measurements (quantitative morphological indicators reflecting horses’ external physical traits) are not limited to conventional indicators such as body weight, body length, wither height and cannon circumference; they also encompass a comprehensive evaluation across multiple dimensions, including skeletal structure, muscle distribution, and locomotion performance [3]. Distinct from other farm livestock, equine wither height serves as a mandatory classification standard for competitive sports grouping worldwide: horses are categorised into ponies (with multiple internal height subgroups), small horses and full-size riding horses according to wither height thresholds. This industry-wide classification requirement creates an urgent practical demand for accurate, automated wither height acquisition, which further highlights the application value of our high-precision three-dimensional (3D) reconstruction measurement system.

Traditional body measurement methods primarily rely on manual operations, using tools such as measuring tapes and callipers to measure animals’ external features directly [4]. Although this approach is relatively simple, it is labour-intensive and highly susceptible to human factors, making it challenging to ensure measurement accuracy and consistency [5,6]. Furthermore, traditional methods have significant limitations in analysing complex body shapes, as they cannot capture the multidimensional phenotypic features required for in-depth research [7]. These shortcomings make traditional methods increasingly unable to meet the demands of modern livestock management for high-precision, large-scale, and objective phenotypic data [8].

With the rapid development of computer vision and artificial intelligence technologies, image-based 3D reconstruction technology has provided a new solution for livestock morphological analysis [9]. Multi-view imaging or Red–Green–Blue Depth (RGB-D) camera technologies can not only capture two-dimensional (2D) image information but also accurately acquire depth data, thereby reconstructing 3D spatial structures [10,11,12,13]. This not only improves measurement accuracy but also significantly enhances efficiency—especially in dynamic phenotypic measurements of moving horses—enabling more stable, reliable results [14].

Despite these advancements, the widespread application of multi-view or RGB-D systems in livestock farming is still hindered by high hardware costs and complex deployment, especially in large-scale or outdoor environments [15]. For equine farming in particular, horses are large, skittish animals that are difficult to restrain for long-duration scanning; most existing 3D measurement systems are designed for confined livestock such as pigs and cattle, and cannot be directly adapted to open barns or equestrian venues. This highlights the necessity of lightweight, image-based 3D reconstruction technology, as 3D generative models are evolving at an unprecedented pace and presenting a promising opportunity for the field of animal 3D reconstruction, which enables 3D reconstruction and body measurement using a small number of ordinary cameras [16].

Owing to its low hardware cost, flexible deployment, and adaptability to complex farming environments, image-based 3D reconstruction technology has attracted extensive attention [17]. This technology aims to recover 3D geometric information about livestock from 2D images, laying the foundation for the automatic extraction of body dimension parameters. However, monocular image reconstruction is inherently a highly ill-posed inverse problem in computer vision. A single 2D image lacks depth information and has inherent depth ambiguity—meaning that the same image may correspond to multiple distinct 3D morphologies and poses. For horses specifically, their elongated trunk, slender limbs, and varied coat patterns further aggravate this ambiguity: limb self-occlusion and uneven surface texture often cause severe local geometric distortions in reconstructed models, especially in the abdomen and cannon bone regions.

Meanwhile, there is a wide variety of livestock species (e.g., cattle, horses, pigs, and sheep), with significant morphological differences among them. Even within the same species, individuals exhibit substantial morphological variation across breeds, growth stages, or behavioural states, which poses significant challenges for accurately inferring their 3D structures from images. Additionally, factors such as lighting changes, occlusions (e.g., fences, feed troughs, and other horses), and complex barn backgrounds further increase the difficulty of reconstruction [18]. To date, most generative image-to-3D methods focus on general objects or companion animals, and few have been validated for on-farm equine body measurement with clinically acceptable accuracy.

To address the challenges mentioned above, researchers have proposed various monocular 3D reconstruction methods for animals, among which template-based methods have emerged as a crucial technical approach due to their unique advantages. Such methods rely on predefined parametric 3D models and reconstruct by matching image features to the models. Skinned Multi-Animal Linear Model (SMAL) [19], a parametric template suitable for quadruped animals, draws its design inspiration from the human SMPL. By estimating shape and pose parameters, it aligns the predefined deformable template with features such as contours and key points in monocular images, generating a 3D model with semantic consistency. Leveraging prior knowledge of animal morphology, template-based methods can effectively alleviate the ambiguity caused by single-view imaging and provide a stable framework for reconstruction. However, such methods also have certain limitations. For instance, SMAL is trained on scanned data of toy animals, resulting in limited ability to represent the morphology of real livestock (e.g., cattle and pigs) [20]. Additionally, the lack of large-scale 3D-annotated datasets for livestock makes it difficult for the model to accurately adapt to the morphological features of different species [21]. To tackle these issues, researchers have continuously improved monocular reconstruction accuracy by introducing species-specific constraints (e.g., cattle’s trunk proportions and pigs’ body shapes) and optimising parametric estimation methods. These efforts also provide valuable insights for the development of monocular reconstruction technology and drive progress in this field [22,23,24,25,26,27].

However, monocular image reconstruction is inherently a highly challenging, ill-posed inverse problem in computer vision. A single 2D image lacks depth information and inherently suffers from depth ambiguity—meaning one image may correspond to multiple distinct 3D morphologies and poses [28]. This depth ambiguity directly leads to the 3D models reconstructed from a single image being unable to accurately represent the proper morphology of livestock, thereby severely compromising the accuracy of body measurement results.

While current technologies for livestock body measurement enable non-contact, high-precision, and high-efficiency measurement, they have been applied in research involving various livestock species. For instance, Du et al. used single-frame point cloud data to measure the body parameters of pigs [29]; Yang et al. completed body dimension evaluation of dairy cows based on 3D point clouds [30]; Luo et al. addressed the interference of pose variations with measurements using statistical shape models [31]. In 2025, Lu et al. proposed a coarse-to-fine automated method for cattle body measurement based on an improved GCN (Graph Convolutional Network) and a 3D parametric model, further enhancing the accuracy and automation of this process [32].

However, improving the accuracy of livestock body measurements while controlling hardware costs and deployment complexity remains a core challenge in the current implementation of precision livestock farming technologies.

Therefore, our study takes horses as the primary research object and proposes a method that combines three-view data with template-based reconstruction technology. By using data from three views (left, right, and top) and leveraging their complementary advantages, the method fundamentally addresses the flaw of missing depth information in a single view. Simultaneously, it avoids the sharp increase in hardware costs caused by excessive view volume. Meanwhile, template-based reconstruction technology can utilise prior knowledge of livestock morphology to achieve accurate image-to-3D reconstruction.

To address the above bottlenecks in equine 3D measurement, this paper clarifies three concise core research objectives integrated with the above technical route:

1. Construct a hSMAL-based decoupled learning pipeline specialised for horse image-to-3D reconstruction [33,34] which effectively mitigates single-view depth ambiguity and realises high-fidelity restoration of full equine morphological structures including trunk contours and limb proportions;
2. Propose a coarse-to-fine non-rigid registration optimisation strategy with dynamic surface feature weights [35] to correct local geometric distortions of reconstructed horse meshes and boost the geometric fitting consistency between mesh models and real horse physiological structures;
3. Build an automated equine body measurement module based on anatomical key point localisation [36] for registration, which can automatically calculate linear body indices and curved circumferential parameters, and open-source the supporting horse dataset and algorithm code for subsequent related equine research.

## 2. Materials and Methods

### 2.1. Experimental Setup and Data Collection

**Figure 1:**
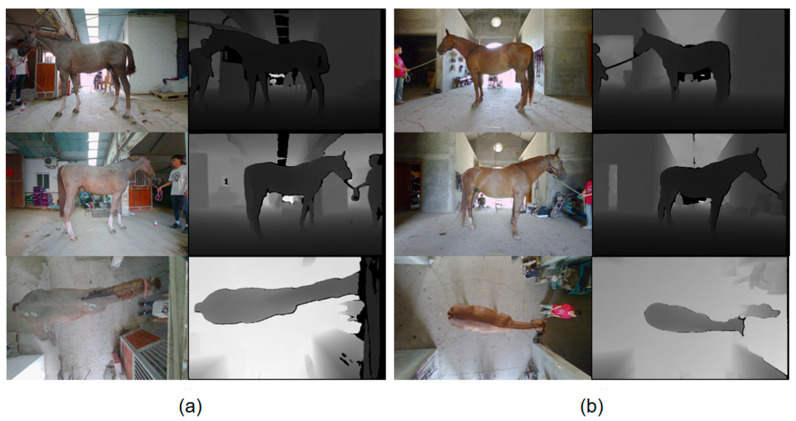
Representative multi-view RGB and depth images of horses from the two datasets. (**a**) Left-view, right-view, and rear-view RGB and depth images of a representative horse from the Zuping dataset. (**b**) Corresponding multi-view RGB and depth images of a representative horse from the Daoxiang Lake dataset.

The experimental dataset used in this study was collected from Zuping International Equestrian Development Co., Ltd., Beijing, China (Zuping), and Daoxiang Lake horse farms, Beijing, China (Daoxiang Lake). It included RGB-D data from three views of 34 horses, as well as manually measured body dimension parameters. Given that this study aims to validate the effectiveness of the proposed technical method rather than drawing population-level conclusions about horses, the sample of 34 horses is appropriate, and coupled with the strictly controlled, high-quality imaging data, it sufficiently supports the statistical analyses of reconstruction accuracy and measurement consistency. During imaging, each horse was led by a staff member to remain stationary at the centre of the camera’s field of view for multi-view data capture. A standard shooting posture was defined to ensure measurement consistency: the horse’s body axis was perpendicular to the left–right-camera axis, the head faced forward naturally, and the limbs stood evenly. The shooting angles were fixed: the left and right cameras were aligned with the horse’s shoulder height, and the top camera was vertically above the horse’s back. RGB images and depth data of the horses were synchronously acquired from three views (left, right, and top) using three ORBBEC Gemini 2-XL cameras (ORBBEC Inc., Shenzhen, China), as shown in Figure 1.

The cameras were installed in a 3D configuration. The collection site had flat terrain and suitable lighting. During camera setup, adjustments were made repeatedly to the height of the top camera and the horizontal distance between the left and right cameras to ensure the entire body of each horse fell within the imaging range.

The top camera was approximately 3.6 m above the central position (where the horse stood). The left and right cameras were each approximately 2.5 m horizontally from the central position, with their heights kept consistent. Once the cameras were correctly set up, a staff member led a horse into the central position from the left and kept it stationary. The cameras then started synchronous imaging, which lasted for 25 s. After imaging was completed, the horse’s name was recorded, and the horse was led out through the right passage. The above process was repeated for the next horse.

**Figure 2:**
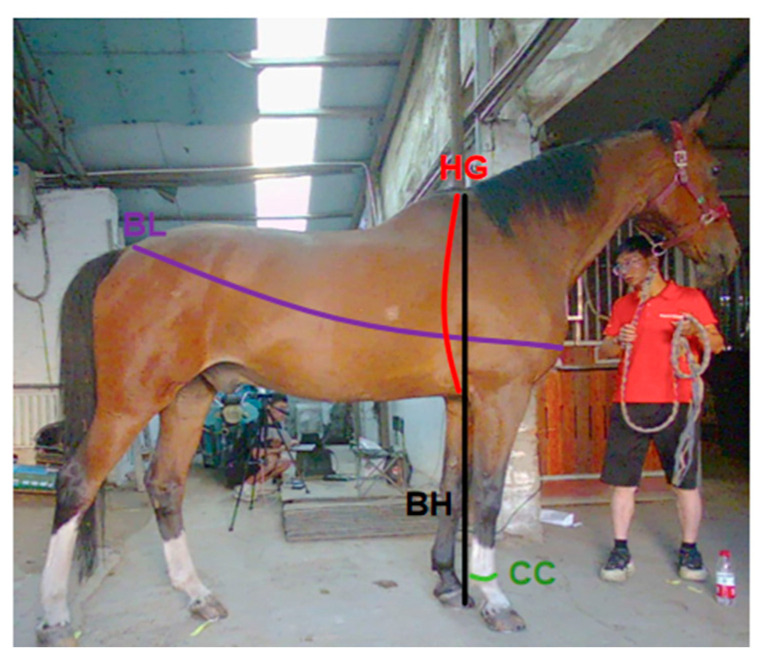
Schematic illustration of the four horse body measurements used in this study. The black line represents body height (BH), measured as the vertical distance from the ground to the highest point of the withers; the purple line represents body length (BL), measured from the point of the shoulder to the posterior point of the hip region; the red line represents heart girth (HG), measured as the circumference of the thoracic region; and the green line represents cannon circumference (CC), measured around the narrowest part of the cannon region of the forelimb.

After camera-based data collection for each horse was finished, manual measurement of the horse’s body dimensions were performed and recorded in detail as shown in Figure 2.

In this research study, data from 34 horses were collected and measured. Four body measurements were manually collected as reference values for evaluating the accuracy of the proposed measurement method, including body height (BH), body length (BL), heart girth (HG), and cannon circumference (CC). The anatomical definitions and manual-measurement procedures were as follows.

Body height (BH) was defined as the vertical distance from the ground to the highest point of the withers. The measurement was taken vertically from the ground surface to the withers. Body length (BL) was defined as the oblique distance from the point of the shoulder to the most posterior point of the hip region. During manual measurement, a flexible measuring tape was positioned along the oblique axis of the body, using the anterior point of the shoulder as the starting landmark and the posterior point of the hip as the endpoint. Heart girth (HG) was defined as the circumference of the thoracic region. It was measured by wrapping a flexible measuring tape around the widest part of the chest and recording the resulting circumference. Cannon circumference (CC) was defined as the circumference of the cannon region of the forelimb. It was measured by wrapping a flexible measuring tape around the narrowest part of the cannon region. Further data details are available in Reference [37].

### 2.2. Data Preprocessing

Data preprocessing is a crucial step in the 3D reconstruction process, and its quality directly affects the accuracy of the final reconstruction results. In this study, preprocessing was performed on 2D horse images and 3D point cloud ground truth to provide accurate input data and ground truth for subsequent experiments [38].

For the 2D horse images used in the reconstruction process, masks were extracted using Grounding DINO and SAM [39,40]. Subsequently, the masked images were cropped to 256 × 256 pixels.

For the 3D point cloud ground truth, to unify the point cloud data from multiple views into a single coordinate system, a rigid registration method was adopted. Since the external parameters of the cameras in this experiment were unknown, CloudCompare 2.13 was used to select 8–12 corresponding points manually. Using these corresponding points, the transformation matrices for the left and right views relative to the top view were calculated separately and applied to subsequent point cloud registration.

**Figure 3:**
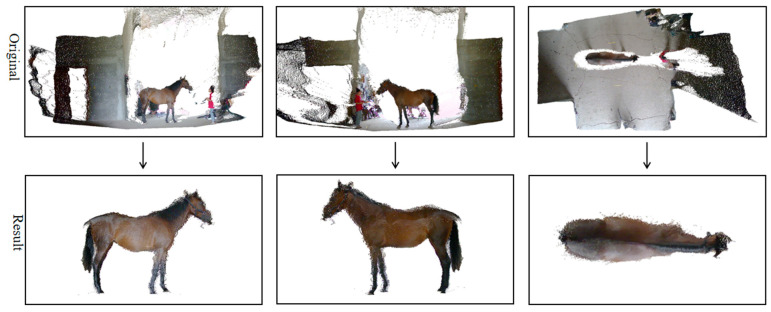
Multi-view point cloud preprocessing results of horses. Each column consists of the raw scene point cloud (**top row**) and the extracted horse point cloud after background removal and denoising (**bottom row**).

To improve the quality of point cloud data and remove unnecessary background information and noise points, threshold segmentation technology was employed for background removal and point cloud denoising. Subsequently, the Random Sample Consensus (RANSAC) algorithm was used for ground detection and segmentation. Finally, the calculated transformation matrices for the three views were applied to all processed point clouds, and batch rigid transformation was performed using the Open3D library in Python 3.8, thereby achieving multi-view point cloud registration. The results of multi-view point cloud processing are shown in Figure 3. Detailed processing steps can be found in the Supplementary Material.

We classify occlusions into three categories and adopt targeted processing strategies:

(1) Background object occlusion (e.g., fences, feed troughs, and barn rails): For image data, we use the SAM segmentation model to generate horse foreground masks and remove background occluders during cropping. For point cloud data, threshold segmentation and RANSAC ground removal further eliminate environmental occlusion noise.
(2) Self-occlusion and partial limb occlusion: Minor partial occlusion is compensated via bilateral view fusion (Section Average Template Computation), whereby geometric information missing in one lateral view can be supplemented by the opposite view. During non-rigid registration, the hSMAL anatomical prior also helps infer reasonable shapes for slightly occluded regions.
(3) Severe occlusion (over 30% of the body area occluded): Samples with severe occlusion are screened out during data preprocessing, as the current template-based framework cannot reliably recover complete shape from heavily occluded inputs.

### 2.3. Experimental Settings

**Figure 4:**
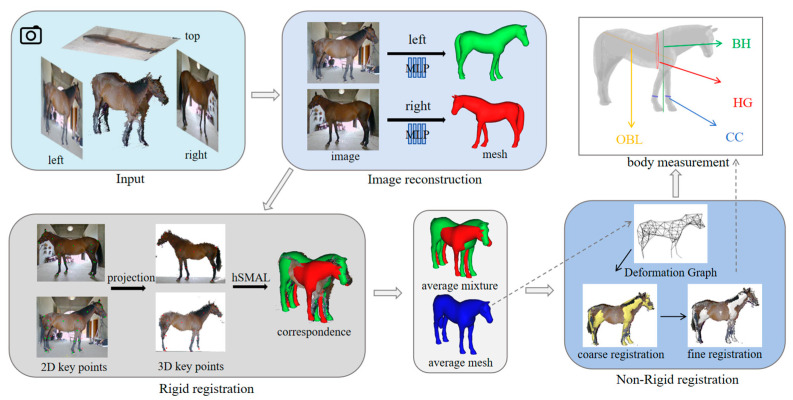
Overall pipeline of the proposed method. The workflow consists of five main stages: multi-view image input, template-based image reconstruction, rigid registration and average mesh fusion, coarse-to-fine non-rigid registration optimisation, and final automated body measurement.

Experiments were conducted following the workflow provided in Figure 4 to realise the reconstruction, optimisation, and measurement of horse images. The image-to-3D inference process was implemented based on the PyTorch 1.11.0 framework on a server equipped with an NVIDIA A40 GPU. The non-rigid registration optimisation method was deployed on a computing environment with the Ubuntu 16 operating system, a 12th Gen Intel(R) Core (TM) i5-12400F CPU processor (2.50 GHz), and 16.0 GB of RAM.

### 2.4. Methods

The overall framework of our method is illustrated in Figure 4. Firstly, in the input stage, multi-view images of the horse (covering left, right, and top perspectives) are acquired to provide high-quality raw data for subsequent processing steps. Subsequently, in the image reconstruction stage, horse mesh models for the left and right views are generated separately from the corresponding images, establishing the initial mapping from 2D images to 3D meshes. Then, the two meshes are fused using a rigid registration technique to generate the average horse mesh. Next, in the non-rigid registration stage, two steps are performed sequentially: coarse registration based on the deformation graph and fine registration. Finally, a precise 3D mesh of the horse is constructed. This mesh can be directly applied to the subsequent horse body dimension measurement process.

#### 2.4.1. Template Mesh Reconstruction Based on Image-to-3D Model

In this experiment, based on a parametric model of horses, Dessie’s encoder–decoder architecture was used to perform 3D reconstruction on an input single-view horse image, inferring a 3D mesh model from 2D images [33].

This framework is selected for two core justifications: first, its decoupled shape–pose estimation strategy effectively disentangles morphological differences from posture variations, which is critical in horses with diverse standing postures in on-barn scenarios; second, it is pre-trained on equine datasets and built upon anatomically consistent mesh topology, avoiding the unrealistic shapes often produced by generic 3D generative models. This experiment implements the extractor–decoder framework combined with the DINO model to infer 3D meshes from input images. It is a multi-stream structure that realises decoupled estimation of shape and pose through an improved contrastive loss $L_{DFL}$. Mathematically, this loss enforces separation between shape and pose feature distributions in the latent space, defined as:

(1) $$L_{DFL}=\omega_{D}\left\{\begin{array}{l} MSE(\gamma_{P_{1}},\gamma_{P_{2}})+MSE(\theta_{G_{1}},\theta_{G_{2}}),if\;appearance\;difference.\\ MSE(\gamma_{A_{1}},\gamma_{A_{2}})+MSE(\theta_{G_{1}},\theta_{G_{2}}),if\;pose\;difference.\\ MSE(\gamma_{A_{1}},\gamma_{A_{2}})+MSE(\gamma_{P_{1}},\gamma_{P_{2}}),if\;global\;rotation\;difference \end{array}\right.$$

where $\omega_{D}$ denotes the balance weight of the loss term; $\gamma_{A}$ and $\gamma_{P}$ represent the appearance latent feature and pose latent feature extracted by the encoder, respectively; and $\theta_{G}$ denotes the global rotation parameter of the horse. When the input sample pair only varies in one single dimension among appearance, pose and global rotation, this loss forces the other two types of features to remain consistent. By minimising this piece-wise loss, the model can establish mutually independent feature subspaces for appearance and pose, eliminating mutual interference between the two types of information during 3D mesh reconstruction. The backbone of our framework adopts the pre-trained DINO-ViT-S8 vision Transformer. In the transfer learning stage, the network freeze the first 12 layers responsible for general visual feature extraction and only fine-tune the last 3 layers to adapt to equine datasets. The input resolution of all images fed into the network is uniformly set to 256 × 256.

In this framework, an extractor based on a Conv2D network is used to extract a single feature representation from the key features of the DINO model’s last layer. The decoder predicts shape parameters β, pose parameters $\theta_{J}$, global rotation parameters $\theta_{G}$, and global translation parameters ξ. In contrast, the extractor $F\left(\cdot \right)$ uses three Conv2D networks on key points to extract features $\gamma_{A}\in \Gamma_{A},\gamma_{P}\in \Gamma_{P}$ and $\gamma_{G}\in \Gamma_{G}$, which correspond to the feature spaces of subject appearance, subject pose, and global information, where $\Gamma_{A},\Gamma_{P},\Gamma_{G}\subseteq R^{640}$.

**Figure 5:**
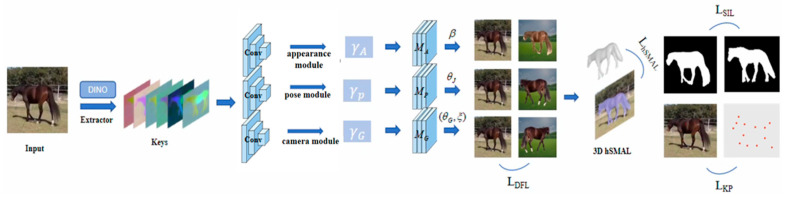
Inference flowchart of the horse image-to-3D reconstruction framework. The framework adopts a multi-stream decoder structure to separately predict shape, pose, and global camera parameters from DINO-extracted image features.

As shown in Figure 5, the decoder consists of three independent sub-modules $M_{A}$, $M_{p}$ and $M_{G}$ each processing a specific set of features extracted by the extractor. Sub-module $M_{A}$ is responsible for inferring shape parameters β from appearance feature $\gamma_{A}$; sub-module $M_{P}$ derives pose parameters $\theta_{J}$ using pose features $\Gamma_{P}$; finally, sub-module $M_{G}$ estimates global rotation parameters $\theta_{G}$ and global translation parameters ξ using global information features $\Gamma_{G}$. The entire framework is composed of an encoder (2) and a decoder (3):

(2) $$\left(\gamma_{A},\gamma_{P},\gamma_{C}\right)=F\left(x\right)$$

(3) $$\beta =M_{A}\left(\gamma_{A}\right),\theta_{J}=M_{P}\left(\gamma_{P}\right),\left(\theta_{G},\xi \right)=M_{G}\left(\gamma_{G}\right)$$

This experiment adopts a weak-perspective camera model $\pi \left(s,t_{x},t_{y}\right)$ which includes a learnable scale s∈R, translation amounts $\left(t_{x},t_{y}\right)\in R^{2}$, and a predefined focal length f = 5000. Based on the relative relationship between the camera and the 3D model, the global translation ξ of the model is defined as shown in (4):

(4) $$\xi =\left[t_{x},t_{y},\frac{2\cdot f}{r\cdot s}\right]$$

where r = 256 is the image resolution. We use a differentiable renderer to project the model back to the image plane, thereby rendering the contour $\overset{‾}{S}$ and 2D key points $\tilde{K}$. By training this learning framework, we can obtain a model capable of inference on images. When applying this model to our horse dataset, we first input the unprocessed horse images directly into the trained model—there is no need for additional complex preprocessing beyond resizing to 256 × 256. The model, trained and inferred on an A100, will automatically use its extractor to pull out appearance, pose, and global features from the input images, then use the decoder to output the 3D horse model’s shape, pose, and global parameters, with an inference time of 9–10 ms per image. Finally, we can visualise the generated 3D horse model directly or use a differentiable renderer to project it onto a 2D plane for comparison with the original image, thereby verifying the inference results.

#### 2.4.2. Scale and Pose Adjustment of Template Meshes Using Rigid Registration

To achieve accurate alignment between reconstruction results and real-acquired RGB-D data in terms of scale and pose, this section focuses on key point extraction and rigid registration and, through their collaboration, achieves precise matching between reconstruction results and real-acquired RGB-D data.

##### Three-Dimensional Correspondence Extraction

In the field of computer vision, key point localisation is a fundamental and critical task. When applied specifically to livestock-related scenarios, accurate key point localisation is a crucial prerequisite for robust rigid registration. This study achieves automated, accurate localisation of key points on horses’ shoulders, hips, and legs, thereby providing reliable point correspondences for the subsequent rigid registration process between the 3D reconstruction results of horses and real-acquired data, and ultimately realises accurate alignment between the two in terms of scale and pose [36].

**Figure 6:**
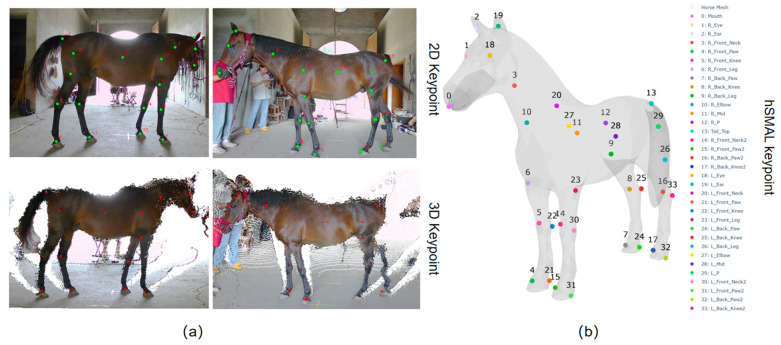
Establishment of semantic key point correspondences for rigid registration. (**a**) Two-dimensional semantic key points are detected from the left- and right-view RGB images using the key point detection model proposed in [36]. The detected 2D key points are then back-projected onto the corresponding 3D point clouds using the depth information and camera calibration parameters to recover their 3D coordinates. (**b**) Corresponding semantic key points are manually defined on hSMAL [34]. These paired key points provide reliable semantic correspondences between the horse model and the acquired 3D point cloud, serving as anchor points for subsequent rigid registration.

As shown in the upper part of Figure 6, the 2D key points detection results for the left and right lateral views of horses are presented from left to right. For the horse dataset used in this study, the focus is primarily on the frontal lateral views (a standardised perspective for livestock body measurement, ensuring consistency in key points positioning across samples). Each lateral view (left and right) contains 18 key points, and the sequence and corresponding names of these key points are detailed in Table 1.

Based on the 2D key points, this section combines the depth images corresponding to the RGB images. By leveraging depth values in the depth images, the 3D spatial coordinates of each key point are calculated from the depth value at the pixel corresponding to that key point and the camera’s intrinsic parameters, enabling conversion from 2D key points to 3D point cloud coordinates.

As shown in Figure 6, the 3D key points are marked in red. During the projection process (conversion from 2D key points to 3D coordinates), the 2D key points corresponding to the horse’s mouth and tail regions each mapped to two distinct 3D positions. To ensure consistency and accuracy of the key points, the average of the two 3D positions was calculated separately for each region, and the resulting average was used to determine the final 3D key point locations. Through this optimisation method, we successfully constructed a set of 34 3D key points for the horse.

##### Rigid Alignment of Reconstructed Models with Point Clouds

To ensure that the reconstructed 3D models are effectively applicable to body measurement, rigid registration between the reconstructed models and the ground truth (manual-measurement-based point clouds) is required to obtain data at a scale consistent with the ground truth. By aligning the key points of the horse’s ground-truth point cloud with those of the parametric reconstructed model, the comparability of measurement data across different horse individuals can be guaranteed.

The reconstruction method adopted in Section 2.4.1 is based on hSMAL and maintains topological consistency. The key points numbered on the hSMAL template can be directly mapped to the reconstructed 3D mesh, with the key point numbering of the mesh being consistent with the sequence and names of the 3D key points described in Section Three-Dimensional Correspondence Extraction.

Rigid registration was then performed between the raw point clouds (P) of 34 horses and the 3D horse meshes (M) generated by the method in Section 2.4.1. First, the data collected by the depth camera were converted from millimetres to metres to ensure consistent data units. Subsequently, the defined key point sets (from the point clouds and from the 3D meshes) were used to perform preliminary spatial alignment. On this basis, the Iterative Closest Point (ICP) algorithm was applied for fine registration to optimise the alignment accuracy between the point clouds and the 3D meshes. Thus, the aligned 3D mesh data were obtained. In this experiment, the number of ICP iterations was set to 150.

##### Average Template Computation

The data used in this experiment originate from horse images captured from three views: left lateral, right lateral, and top. Due to limitations in single-view reconstruction, local geometric features may deviate (e.g., obscured limb details or distorted trunk contours caused by occlusions or perspective bias).

Fusing the geometric information from the left and right lateral view meshes can effectively balance feature representation across different views: the left view compensates for occlusions on the right side of the horse and vice versa, and vertex-wise averaging suppresses random reconstruction noise from individual views. Compared with using a single view, this bilateral fusion strategy improves the stability of the base mesh without introducing additional computational overhead. We adopted a geometric fusion strategy based on vertex-weighted averaging. Leveraging the linear fusion property of vertex coordinates across homologous topological meshes, we assigned equal weights (0.5:0.5) to the left and right meshes. We computed the weighted average of the corresponding vertices’ coordinates. On the premise of maintaining the topological structure, this approach integrates geometric information from the two lateral views, balances the reconstruction errors of single-view data, and generates a fused mesh that incorporates features from both sides, thereby providing a more robust base model for subsequent fitting. We use this average model as the source model.

#### 2.4.3. Non-Rigid Fitting Optimisation

**Figure 7:**
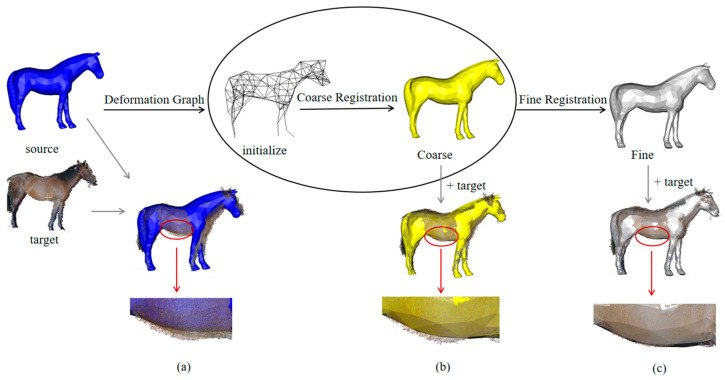
Workflow of the coarse-to-fine non-rigid registration optimisation. (**a**) Source mesh (blue) and target point cloud (brown, ground-truth point cloud) before registration, showing initial local misalignment. (**b**) Mesh after coarse registration (yellow), showing improved global alignment. (**c**) Mesh after fine registration (grey), showing refined local geometric details.

Using the reconstruction method outlined in Section 2.4.1, we obtained favourable reconstruction results from horse images. However, both quantitative and qualitative analyses in Section 3.2 reveal some discrepancies between these results and the ground truth. Direct one-step fine non-rigid registration is prone to falling into local optima when the initial mesh has large pose or scale deviations and may even produce topologically unreasonable deformations on slender limbs. To address this, we design a coarse-to-fine non-rigid registration pipeline for reconstruction optimisation, built upon the average mesh from Section Average Template Computation. The coarse stage first achieves global alignment via a deformation graph, while the fine stage refines local geometric details; this hierarchical strategy balances registration robustness and geometric accuracy and is particularly effective in correcting the limb distortions common in image-based horse reconstruction [35]. The overall workflow is illustrated in Figure 7. Detailed non-rigid registration steps can be found in the Supplementary Material.

##### Coarse Registration

A good initialisation is crucial to achieving desirable registration results for the local solver. In this section, a coarse registration stage based on a deformation graph is introduced prior to fine registration. The horse deformation graph is constructed as a graph structure $G=\{V_{G},E_{G}\}$, where the node subset $V_{G}=\{p_{1},\ldots ,p_{\left|V_{G}\right|}\}$ is uniformly sampled from the average model mesh vertices, and the edge set $E_{G}$ is formed by connecting the neighbouring nodes. Each node $p_{j}$ is associated with an affine transformation, represented by a matrix $A_{j}\in R^{3\times 3}$ and a vector $t_{j}\in R^{3}$. The deformed position of a source point $v_{i}$ is determined by a weighted combination of its influential node set $I(v_{i})=\{p_{j}\in V_{G}|D(v_{i},p_{j})<R\}\;where\;D(\cdot ,\cdot )$ denotes the geodesic distance, specifically given by Equation (5):

(5) $${\hat{v}}_{i}=\sum_{p_{j}\in I\left(v_{i}\right)}\;w_{ij}\cdot \left[A_{j}\left(v_{i}-p_{j}\right)+p_{j}+t_{j}\right]$$

Here, the influence weight is defined as (6):

(6) $$w_{ij}=\frac{(1-D^{2}(v_{i},p_{j})/R^{2}{)}^{3}}{\sum_{p_{k}\in I\left(v_{i}\right)}\;(1-D^{2}(v_{i},p_{k})/R^{2}{)}^{3}}$$

To achieve coarse alignment, the optimisation objective function includes an alignment term $E_{align}^{C}$ and an ARAP (As-Rigid-As-Possible) term $E_{ARAP}^{C}$ like Equation (7). For computational efficiency, the alignment term is calculated only on a sampled subset of the source points:

(7) $$E_{align}^{C}=\frac{1}{\left|S\right|}\sum_{v_{s}\in S}\;E_{align}^{s}$$

Additionally, two regularisation terms are introduced with the aim of ensuring smooth deformation during the coarse registration process, thereby preserving the structural integrity of the source mesh following Equation (8).

(8) $$E_{smo}=\frac{1}{2\left|E_{G}\right|}\sum_{p_{i}\in V_{G}}\;\sum_{p_{j}\in N\left(p_{i}\right)}\;∥D_{ij}{∥}^{2}$$

where $D_{ij}=r_{ij}[A_{j}(p_{i}-p_{j})+p_{j}+t_{j}-(p_{i}+t_{i})]$ and $r_{ij}$ is a normalised weight; the entire smoothness term is used to control the transformation difference between adjacent graph nodes (Formula (9)).

(9) $$E_{rot}=\frac{1}{\left|V_{G}\right|}\sum_{p_{j}\in V_{G}}\;∥A_{j}-{proj}_{R}\left(A_{j}\right){∥}_{F}^{2}$$

Here, ${proj}_{R}(\cdot )$ projects a matrix onto its nearest rotation matrix, compared with the ARAP term, this term maintains local rigidity at a larger scale. Each term in the objective serves a clear mathematical and physical purpose:

$E_{align}^{C}$: Data term that drives source mesh vertices toward their corresponding target points, measuring geometric fitting accuracy.

$E_{ARAP}^{C}$: As-Rigid-As-Possible regularisation that preserves local surface rigidity, preventing unnatural mesh stretching.

$E_{smo}$: Smoothness term that penalises abrupt transformation differences between adjacent graph nodes, ensuring globally continuous deformation.

$E_{rot}$: Rotation regularisation that constrains affine transforms to be close to pure rotations, eliminating shearing or scaling artifacts.

The final optimisation problem is formulated as (10):

(10) $$\underset{\left\{\left(A_{j},t_{j}\right)\right\},\left\{R_{i}\right\}}{min}\;E_{align}^{C}+w_{ARAP}^{C}E_{ARAP}^{C}+w_{smo}E_{smo}+w_{rot}E_{rot}$$

The resulting coarse alignment serves as the initial input for subsequent fine registration, significantly enhancing the algorithm’s robustness and convergence.

##### Fine Registration

After completing coarse registration via the deformation graph in Section Coarse Registration and obtaining good initial alignment, fine registration performs refined non-rigid registration on this basis to achieve higher-precision alignment. In this stage, the deformed positions $\left\{{\hat{v}}_{i}\right\}$ of each source point are directly optimised. An alternating optimisation strategy is adopted to solve the complete objective function, which includes a Symmetric Point-to-Plane (SP2P) alignment term and an ARAP regularisation term, as shown in Equation (11):

(11) $$\underset{\left\{{\hat{v}}_{i}\right\},\left\{R_{i}\right\}}{min}\;E_{align}+w_{ARAP}E_{ARAP},s.t.R_{i}^{T}R_{i}=I,det\left(R_{i}\right)=1,\forall i$$

Specifically, in the (k + 1)-th iteration, the algorithm updates the following variables sequentially.

Nearest-point correspondences $\{u_{\rho_{i}}\}:$ Fix the current deformed point positions ${\hat{v}}_{i}^{\left(k\right)}$, and find the nearest point in the target point cloud for each source point, following Equation (12):

(12) $$\rho_{i}^{\left(k+1\right)}=\arg\underset{\rho_{i}\in \left\{1,\ldots ,\left|U\right|\right\}}{min}\;∥u_{\rho_{i}}-{\hat{v}}_{i}^{\left(k\right)}∥$$

Subsequently, update the robust weights $\left\{\alpha_{i}^{\left(k+1\right)}\right\}$ based on $\alpha_{i}$. Weight $\alpha_{i}$ is computed based on the deformed position ${\hat{v}}_{i}^{\prime }$, the corresponding point $u_{\rho_{i}^{\prime }}$, and their normals ${\hat{n}}_{i}^{\prime }$, $n_{\rho_{i}^{\prime }}^{t}$ in the previous iteration, following Equation (13):

(13) $$\alpha_{i}=\left\{\begin{array}{l} 0,\;\;\;\;\;\;\;\;\;\;\;\;\;\;\;\;\;\;\;\;\;\;\;\;\;\;\;\;\;\;\;\;\;\;\;\;\;{\hat{n}}_{i}^{\prime }\cdot n_{\rho_{i}^{\prime }}^{t}<0,\\ exp(-\frac{∥{\hat{v}}_{i}^{\prime }-u_{\rho_{i}^{\prime }}{∥}^{2}}{2\sigma^{2}}),\;\;\;\;otherwise\;\;\;\; \end{array}\right.$$

Position variables $\left\{{\hat{v}}_{i}\right\}$: Fix the nearest points $\left\{u_{\rho_{i}^{\left(k+1\right)}}\right\}$, rotation matrices $\left\{R_{i}^{\left(k\right)}\right\}$, and weights $\left\{\alpha_{i}^{\left(k+1\right)}\right\}$ and then update $\left\{{\hat{v}}_{i}\right\}$ by solving a sparse linear system, following Equation (14):

(14) $$\left(\frac{N^{T}W^{T}WN}{\left|V\right|}+\frac{w_{ARAP}B^{T}B}{2\left|E\right|}\right){\hat{V}}^{\left(k+1\right)}=\frac{N^{T}W^{T}WT}{\left|V\right|}+\frac{w_{ARAP}B^{T}Y}{2\left|E\right|}$$

Since the system matrix is sparse, symmetric, and positive definite, Cholesky decomposition is used for solving.

Rotation matrix variables $\left\{R_{i}\right\}$: Fix $\left\{{\hat{v}}_{i}^{\left(k+1\right)}\right\}$ and $\left\{u_{\rho_{i}^{\left(k+1\right)}}\right\}$, and solve the rotation optimisation subproblem independently for each $\left\{R_{i}\right\}$ following Equation (15):

(15) $$\begin{array}{cc} \underset{R_{i}}{min}\;\alpha_{i}^{\left(k+1\right)}[(R_{i}n_{i} & {+n_{\rho_{i}^{\left(k+1\right)}}^{t})\cdot ({\hat{v}}_{i}^{\left(k+1\right)}-u_{\rho_{i}^{\left(k+1\right)}})]}^{2}+\omega \sum_{v_{j}\in N(v_{i})}\;\\ & ∥\left({\hat{v}}_{i}^{\left(k+1\right)}-{\hat{v}}_{j}^{\left(k+1\right)}\right)-R_{i}\left(v_{i}-v_{j}\right){∥}^{2},s.t.R_{i}^{T}R_{i}=I,det\left(R_{i}\right)=1 \end{array}$$

where $\omega =\frac{w_{ARAP}\cdot |V|}{\left|N(v_{i})|\cdot 2|E\right|}$. To update $R_{i}$, we adopt the MM (Majorization–Minimization) strategy and minimise a surrogate function, with further details referred to the original paper.

#### 2.4.4. Automated Body Measurement

Accurate measurement of horse body dimensions is a crucial method for evaluating horses’ health, athletic performance, and breed characteristics [41]. By collecting and analysing horse body dimension data, valuable information can be provided for horse conformation assessment, training, and feeding management.

In this research study, data from 34 horses were collected and measured. The measurements are relatively comprehensive, with a primary focus on four key indicators: body height (BH), body length (BL), heart girth (HG), and cannon circumference (CC). These indicators can effectively reflect the conformation characteristics and health status of horses. The measurement positions of these four indicators on the corresponding horse are shown in Figure 2.

For linear measurement parameters such as body height and body length, we adopt the spatial Euclidean distance calculation method. Let the coordinates of two points be MP_1_ (x_1_, y_1_, z_1_) and MP_2_ (x_2_, y_2_, z_2_), respectively. The Euclidean distance (straight-line distance) between the two points can be calculated using Formula (16):

(16) $$D=\sqrt{{\left(x_{2}-x_{1}\right)}^{2}+{\left(y_{2}-y_{1}\right)}^{2}+{\left(z_{2}-z_{1}\right)}^{2}}$$

To calculate heart girth and cannon circumference, we propose a method to compute the perimeter of specific cross-sections on the 3D model based on key point definition and mesh cutting technology. For the 3D key points defined on the horse mesh, PCA is used to fit an optimal plane. The mathematical foundation of this method is that the optimal fitting plane of a set of 3D points is perpendicular to the direction of minimum spatial variance. For the 3D key points $P_{h}=\{p_{1},p_{2},\ldots ,p_{m}\},$ defined on the horse mesh, principal component analysis (PCA) is employed to solve for this plane. We first compute the centroid of the point set and construct the covariance matrix as described in Formula (17):

(17) $$\Sigma =\frac{1}{m}{\sum_{i=1}^{m}\left(p_{i}-\bar{p}\right)(p_{i}-\bar{p})}^{T}$$

where $\bar{p}$ is the centroid. Eigenvalue decomposition of Σ yields three orthogonal eigenvectors; the eigenvector corresponding to the smallest eigenvalue is taken as the unit normal vector n of the fitting plane.

This plane is defined by its unit normal vector n and offset distance d from the origin, as described in Equation (18):

(18) n⋅x+d=0

where $x\in R^{3}$ is any point on the plane.

First, calculate the centroid of the key point set $\bar{p}=\frac{1}{m}\sum_{i=1}^{m}\;p_{i}$ and centralise the point set. Then perform eigenvalue decomposition on the covariance matrix. The normal vector n corresponds to the eigenvector associated with the smallest eigenvalue, i.e., the direction with the smallest data variation. The offset d is calculated by Formula (19):

(19) $$d=-n\cdot \bar{p}$$

To extract the cross-sectional contour (for heart girth and cannon circumference measurement), the fitted plane is intersected with the triangular mesh surface. In the process of intersecting the plane with the triangular mesh, it is necessary to calculate the intersection point between each triangular edge and the plane. The position of this intersection point can be determined by weighing the endpoints’ coordinates and their distances from the plane. $p_{int}$ is the position of the intersection point, and d_i_ and d_j_ are the distances from vertices v_i_ and v_j_ to the plane, respectively (Formula (20)):

(20) $$p_{int}=v_{i}+\frac{-d_{i}}{d_{j}-d_{i}}\left(v_{j}-v_{i}\right)$$

Subsequently, for each generated polyline $L=\{p_{1},p_{2},\ldots ,p_{n}\}$, calculate the Euclidean distance $∥p_{i+1}-p_{i}∥$ between adjacent intersection points on it, and sum the lengths of all line segments to obtain the total length of the polyline. Finally, the complete cross-sectional perimeter is obtained as $L_{total}=\sum_{i=1}^{n-1}\;∥p_{i+1}-p_{i}∥$, thereby realising the automatic perimeter measurement of heart girth and cannon circumference on the 3D horse mesh.

#### 2.4.5. Three-Dimensional Evaluation Metrics

This paper employs four quantitative evaluation metrics to assess the performance of the 3D horse reconstruction model, covering both geometric fitting accuracy and feature capture integrity. These metrics, along with their core functions and experimental roles, are detailed as follows.

Chamfer Distance (CD), Precision, Recall, and F-Score are employed as evaluation metrics. These metrics are used to comprehensively assess the geometric differences between the ground-truth point cloud and the reconstructed mesh, as well as the reconstruction’s coverage.

Chamfer Distance is utilised to assess the geometric similarity between two point clouds according to Equation (21). A smaller CD value indicates a higher degree of geometric shape matching between the reconstructed mesh and the ground-truth point cloud.

(21) $$CD\left(P,Q\right)=\frac{1}{N}\sum_{i=1}^{N}\underset{j}{min}∥p_{i}-q_{j}{∥}_{2}^{2}+\frac{1}{M}\sum_{j=1}^{M}\underset{i}{min}∥q_{j}-p_{i}{∥}_{2}^{2}$$

Precision measures the proportion of valid components in the reconstructed point cloud according to Equation (22). A higher precision value signifies better reconstruction results.

(22) $$\mathrm{Precision}@\tau =\frac{\left|\left\{p\in P_{recon}|\exists \;q\in P_{gt},∥p-q∥<\tau \right\}\right|}{\left|P_{recon}\right|}\times 100\%$$

Recall reflects the extent to which the reconstruction results cover the real object surface according to Equation (23). A higher Recall value indicates a more complete reconstruction with fewer missing parts.

(23) $$Recall@\tau =\frac{\left|\left\{q\in P_{gt}|\exists \;p\in P_{recon},∥q-p∥<\tau \right\}\right|}{\left|P_{gt}\right|}\times 100\%$$

F-Score is the harmonic mean of Precision and Recall according to Equation (24). It comprehensively considers both the accuracy and completeness of the reconstruction, serving as a balanced metric for evaluating the overall reconstruction quality.

(24) $$\text{F-Score}@\tau =\frac{2\cdot Precision@\tau \cdot Recall@\tau }{Precision@\tau +Recall@\tau }$$

## 3. Results

### 3.1. Dataset for Result Analysis

All experiments were performed on the multi-view RGB-D horse dataset introduced in Section 2.1, which contains 34 horse individuals collected from Zuping and Daoxiang Lake equestrian farms with diverse breeds and age distributions. The dataset is split into two subsets: the Zuping subset with 16 horses and the Daoxiang Lake subset with 18 horses. Compared with the well-controlled shooting environment at Zuping farm, samples from Daoxiang Lake exhibit greater variations in horse standing poses and on-site lighting conditions. For all subsequent qualitative visualisation and quantitative metric analysis, we only utilised specimens with intact point cloud data and stable convergence of the non-rigid registration pipeline.

### 3.2. Evaluation from Perspective of 3D Reconstruction

#### 3.2.1. Three-Dimensional Horse Body Reconstruction Results

**Figure 8:**
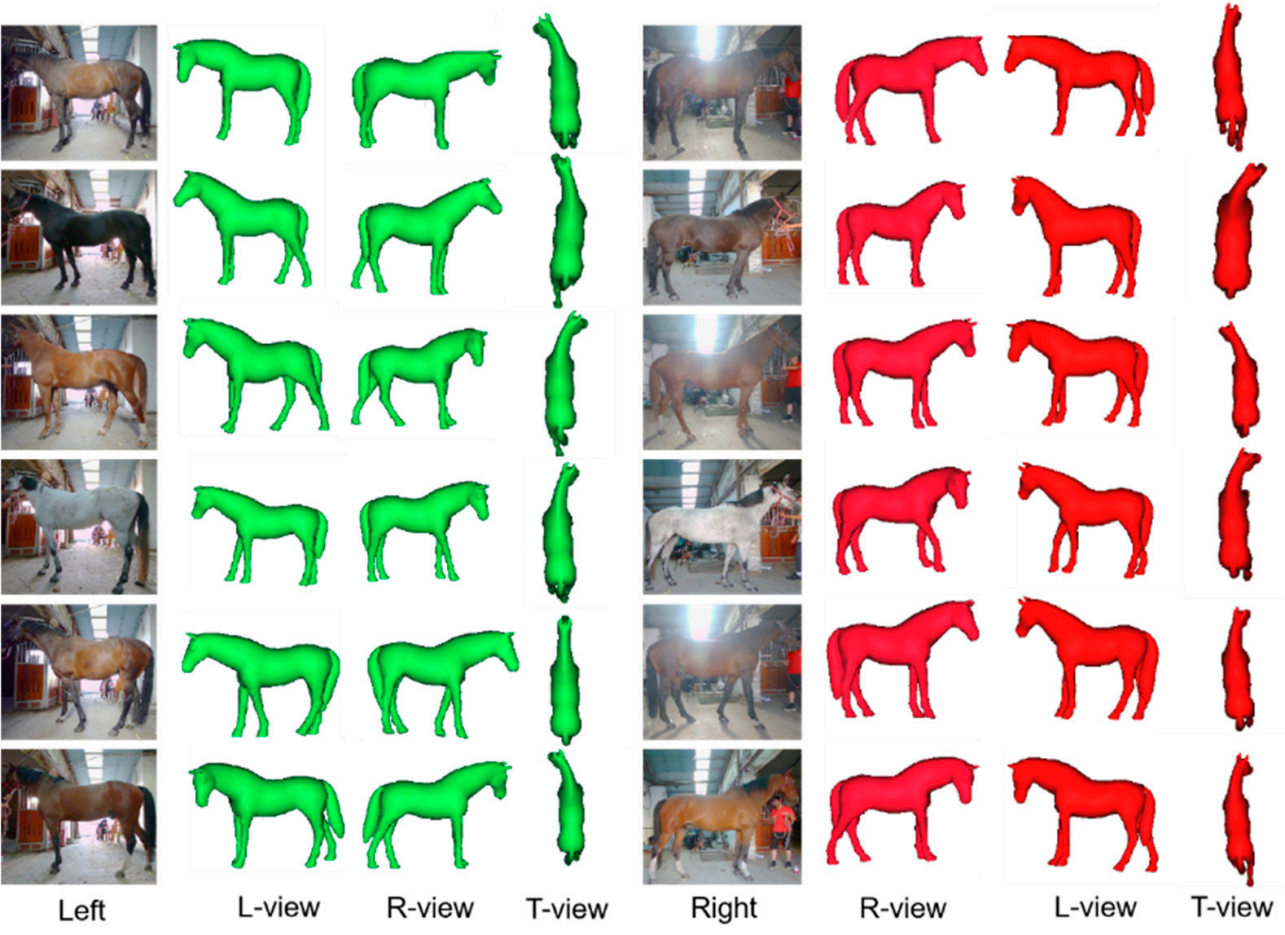
Initial single-view 3D reconstruction results for the same group of horses. The green models were reconstructed from single left-view images, whereas the red models were reconstructed from single right-view images. Each reconstructed model is visualised from three different viewpoints for comparison.

Using the single-view image reconstruction method in Section 3.1, we completed the separate reconstruction of the left and right images from Zuping and Daoxiang Lake. To clearly demonstrate the reconstruction effects and viewing-angle differences for the same horse sample, we have presented visualisations of its initial reconstruction results from three views in Figure 8.

As shown in Figure 8, the reconstructed models generally preserve the overall body shape and major anatomical structures of the corresponding horses. The multi-view visualisations further allow the reconstructed geometry to be qualitatively assessed from different viewing directions.

#### 3.2.2. Average (Source) Template Results

Although the single-view reconstruction results obtained from the left- and right-view images were generally consistent in overall pose and body shape, small differences remained in local geometric details. To reduce the influence of view-specific reconstruction variations and obtain a more stable initial model for the subsequent processing stage, the left- and right-view-reconstructed meshes were fused using the averaging strategy described in Section Average Template Computation.

**Figure 9:**
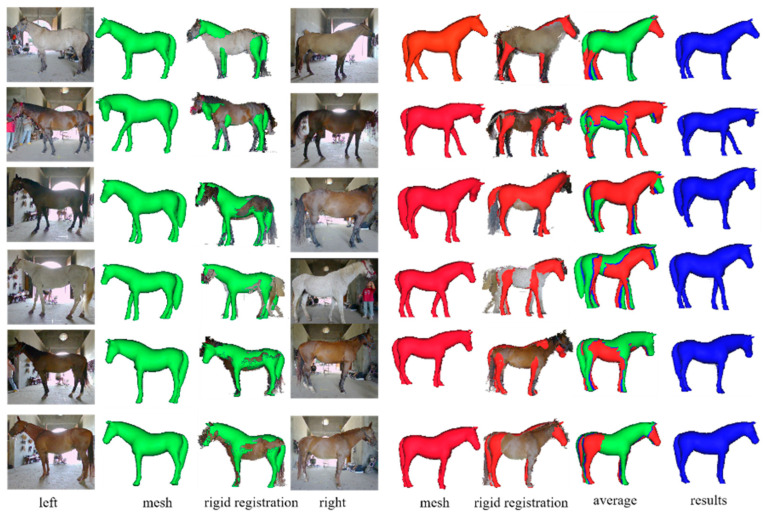
Representative results of left–right view mesh averaging and fusion. From left to right, the columns show the left-view RGB image, the corresponding single-view-reconstructed mesh (green), and its rigid registration with the acquired 3D point cloud, followed by the right-view RGB image, the corresponding single-view-reconstructed mesh (red), and its rigid registration result. The aligned left- and right-view-reconstructed meshes are subsequently averaged to generate the final fused mesh, shown in blue.

Although the models reconstructed from the left- and right-view images were generally similar in overall pose and body shape, small differences remained in local geometric details. Therefore, after rigid registration with the acquired 3D point cloud, the aligned left- and right-view-reconstructed meshes were averaged to reduce view-dependent reconstruction variation and generate a fused mesh for subsequent processing. Representative results are shown in Figure 9.

#### 3.2.3. Non-Rigid Fitting Results

**Figure 10:**
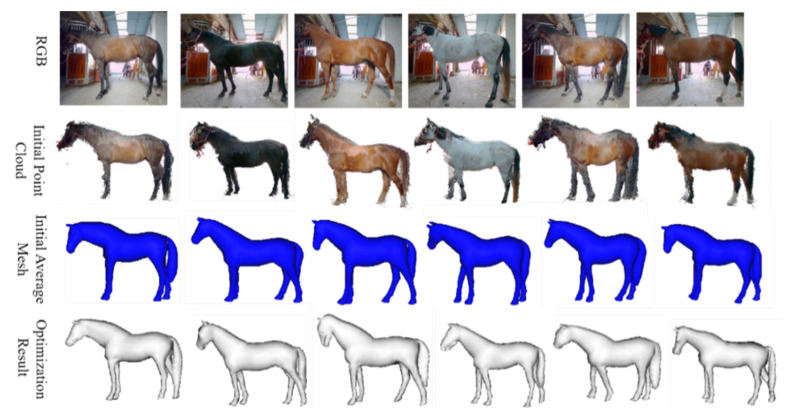
Qualitative comparison of final reconstruction results. From left to right: input RGB image, ground-truth point cloud, initial average mesh, and optimised mesh after non-rigid registration. The optimised mesh shows significantly better alignment with the real horse contour, especially in the limb and abdomen regions.

For the final reconstruction results obtained via non-rigid optimisation in Section 2.4.3, the ground truth and the initial average model were also introduced as references for comparative analysis, and the specific comparison of partial reconstruction results is shown in Figure 10.

#### 3.2.4. Quantitative Assessment of Non-Rigid Registration via 3D Metrics

This subsection adopts the 3D evaluation metrics proposed in Section 2.4.5, whose mathematical formulations are shown in Equations (21)–(24). We utilise these unified indicators to conduct a quantitative comparison between the averaged mesh model and the non-rigid registration approach on the Daoxiang Lake and Zuping datasets, and the detailed numerical results are summarised in Table 2.

Table 2 summarises the quantitative comparison between the averaged meshes and the non-rigid registration results on the Daoxiang Lake and Zuping datasets. Overall, the non-rigid registration results showed improved geometric alignment with the acquired 3D point clouds, although the degree of improvement varied across metrics and datasets.

For the Daoxiang Lake dataset, the model optimised via non-rigid registration (non-rigid (d)) exhibits significant improvements in all metrics compared with the average model (average (d)): Chamfer Distance decreases from 11.46 cm to 5.06 cm, representing a substantial reduction in the geometric deviation between the model and the real target. Precision increases from 62.63% to 87.70%, and Recall rises from 52.95% to 83.78%, indicating a marked enhancement in the model’s ability to capture target features and the completeness of feature extraction; correspondingly, the F-Score—which comprehensively reflects Precision and Recall—increases from 57.14% to 85.54%, verifying the optimisation effect of non-rigid registration on the overall performance of the model.

In the Zuping dataset, the optimisation effect of non-rigid registration is equally significant: the Chamfer Distance drops from 10.858 cm to 5.362 cm, leading to a noticeable improvement in geometric fitting accuracy; Precision increases from 66.12% to 88.24%, and Recall climbs from 52.33% to 77.87%, resulting in better accuracy and comprehensiveness of feature extraction; the F-Score also increases from 58.33% to 82.70%. Consistent results across the two datasets demonstrate that the non-rigid registration method effectively reduces the geometric error of the model, improves feature matching performance, and demonstrates good adaptability across datasets.

#### 3.2.5. Evaluation from the Perspective of Body Measurement

##### Body Measurement Results

During the experimental data preprocessing stage, screening was conducted based on quality differences across different datasets. Among them, due to the poor quality of data acquisition for some original data in the Daoxiang Lake dataset, two main issues arose: first, the quality of the generated original point clouds was unsatisfactory, and second, there was a lack of real measurement indicators. This defect directly affected the subsequent non-rigid registration process: the inability to accurately calculate the distance constraints between feature points ultimately led to the convergence failure of the iterative optimisation process. Therefore, to ensure the reliability of experimental results, six horse samples in this dataset were excluded from the subsequent quantitative analysis. In contrast, the Zuping dataset underwent more rigorous control during the original data acquisition process. Both the data integrity and accuracy met the experimental requirements, so all samples were used for subsequent analysis. Accordingly, the measurement results of horses from the Daoxiang Lake dataset and Zuping dataset—including GT (ground truth), average (average template results), and non-rigid (fitting results)—are provided in Table 3 and Table 4, respectively.

##### Body Measurement Accuracy Comparison

In this section, Mean Absolute Error (MAE) and Mean Absolute Percentage Error (MAPE) are adopted as the evaluation indicators for body measurements according to Equations (25) and (26). Among them, MAE can reflect the magnitude of the deviation between the estimated values and the true values, while MAPE can reflect the estimation accuracy of each body measurement indicator and the overall accuracy of the method.

(25) $$MAE=\frac{1}{n}\sum_{i=1}^{n}\left|y_{i}-{\hat{y}}_{I}\right|$$

(26) $$MAPE=\frac{1}{n}\sum_{i=1}^{n}\left|\frac{y_{i}-{\hat{y}}_{i}}{y_{i}}\right|\times 100\%$$

**Figure 11:**
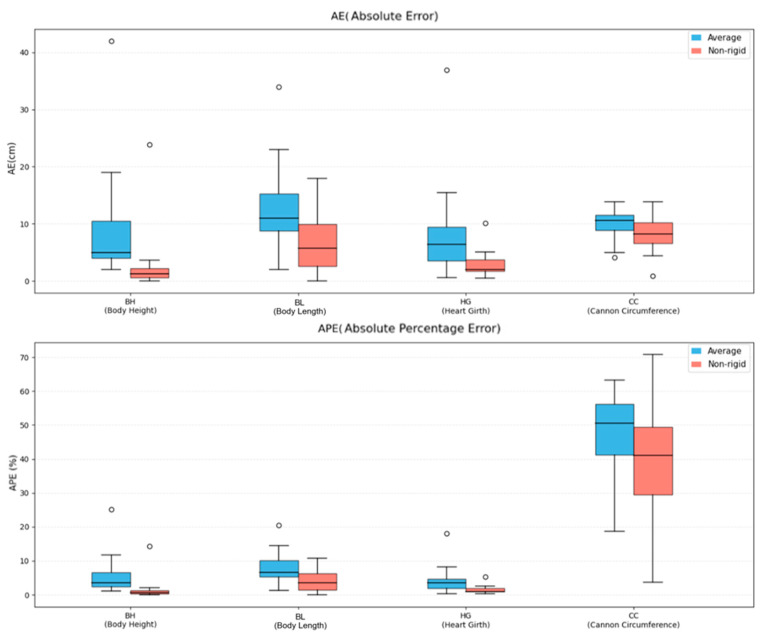
Distributions of body measurement errors for the Daoxiang Lake dataset. The box plots compare the average and non-rigid results for four body measurements: body height (BH), body length (BL), heart girth (HG), and cannon circumference (CC). The upper panel shows the distributions of absolute error (AE, cm), whereas the lower panel shows the distributions of absolute percentage error (APE, %). Each observation represents the measurement error of an individual horse. Boxes represent the interquartile range (Q1–Q3), the horizontal line within each box indicates the median, whiskers extend to the most extreme observations within 1.5 times the interquartile range, and circles indicate outliers.

**Figure 12:**
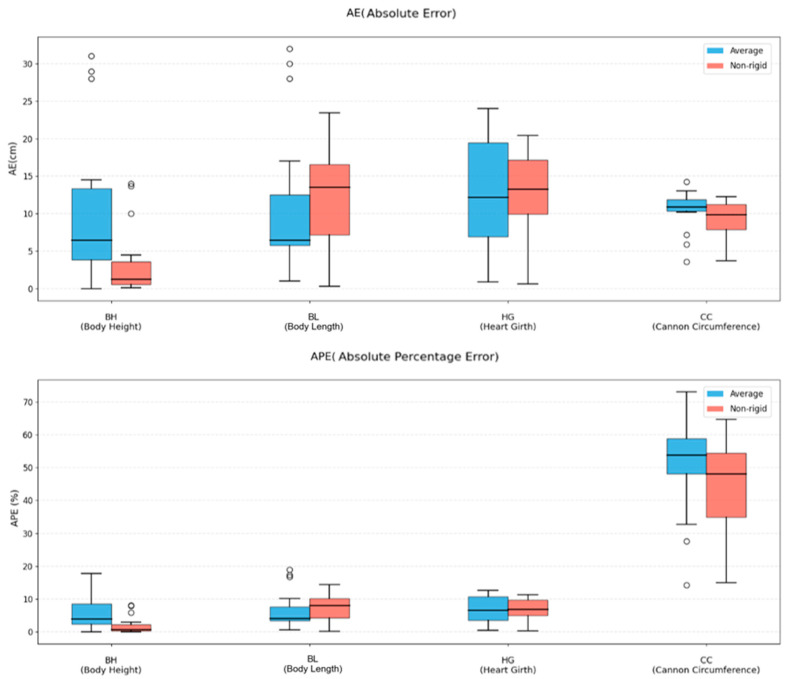
Distributions of body measurement errors for the Zuping dataset. This figure presents box plots of errors (for average and non-rigid categories) across four body measurements (body height (BH), body length (BL), heart girth (HG), cannon circumference (CC)) in the Zuping dataset, via two metrics: Absolute Error (AE, cm, upper subplot) and Absolute Percentage Error (APE, %, lower subplot). Here, boxes represent interquartile ranges (Q1–Q3), whiskers denote normal fluctuation ranges (1.5× interquartile range), and circles indicate outliers.

Figure 11 and Figure 12 visualise the sample-wise error distributions of the proposed reconstruction method on the Daoxiang Lake and Zuping datasets across four anatomical measurements (body height (BH), body length (BL), heart girth (HG), and cannon circumference (CC)). The box plots illustrate raw Absolute Error (AE, cm) and Absolute Percentage Error (APE, %) for each horse specimen within both datasets, revealing the median, dispersion and outliers of per-sample prediction errors.

Table 5 and Table 6 present the quantitative evaluation results of the proposed reconstruction method on the Daoxiang Lake and Zuping datasets, respectively, with two key metrics—Mean Absolute Error (MAE, in cm) and Mean Absolute Percentage Error (MAPE, in %)—used to assess performance across four anatomical measurements: body height (BH), body length (BL), heart girth (HG), and cannon circumference (CC).

For the Daoxiang Lake dataset (Figure 11, Table 5), the non-rigid model demonstrates significant performance improvements over the average model. In terms of MAE, the non-rigid model reduces errors by a relative 63.59% (from 9.20 cm to 3.35 cm) for BH, a relative 49.23% (from 13.06 cm to 6.63 cm) for BL, a relative 63.60% (from 8.49 cm to 3.09 cm) for HG, and a relative 16.58% (from 9.71 cm to 8.10 cm) for CC. Similarly, the MAPE of the non-rigid model is lower than that of the average model across all metrics: a relative 64.44% reduction for BH (from 5.68% to 2.02%), a relative 48.77% reduction for BL (from 8.10% to 4.15%), a relative 63.70% reduction for HG (from 4.38% to 1.59%), and a relative 15.74% reduction for CC (from 46.44% to 39.13%).

For the Zuping dataset (Figure 12, Table 6), the non-rigid model also outperforms the average model in most metrics, confirming the method’s generalisability. In MAE, the non-rigid model achieves a relative 67.82% reduction for BH (from 10.69 cm to 3.44 cm), while for CC, it reduces the error by a relative 9.94% (from 10.46 cm to 9.42 cm). For MAPE, the non-rigid model shows a relative 68.32% decrease for BH (from 6.47% to 2.05%) and a relative 10.09% decrease for CC (from 51.05% to 45.9%). Although slight increases in MAE and MAPE are observed for BL and HG in the Zuping dataset, the overall error reduction across critical metrics (BH and CC) still validates the effectiveness of the non-rigid optimisation approach in enhancing reconstruction accuracy. The steps for body measurement and 3D evaluation are presented in the Supplementary Material.

#### 3.2.6. F-Score-Based Quantitative and Visual Analyses Under Different Reconstruction Quality Conditions

As introduced in Section 2.4.5, the F-Score provides an integrated measure of geometric agreement by balancing Precision and Recall. In the proposed pipeline, non-rigid registration is initialised from the multi-view averaged mesh generated from the left- and right-view reconstructions. Therefore, the geometric quality of the averaged mesh is an important factor affecting the subsequent non-rigid alignment performance. To further analyse the influence of initial reconstruction quality, we stratified the valid samples according to the F-Score of the averaged mesh before non-rigid registration.

In practical on-barn image acquisition, some horses naturally stand in skewed, rotated, or asymmetric postures. Under such conditions, the left and right views may capture different body outlines, leading to inconsistent single-view reconstructions. When these inconsistent reconstructions are averaged, local or global geometric deviations may appear in the resulting averaged mesh. Such deviations can further affect the reliability of the initial mesh used for non-rigid registration.

**Figure 13:**
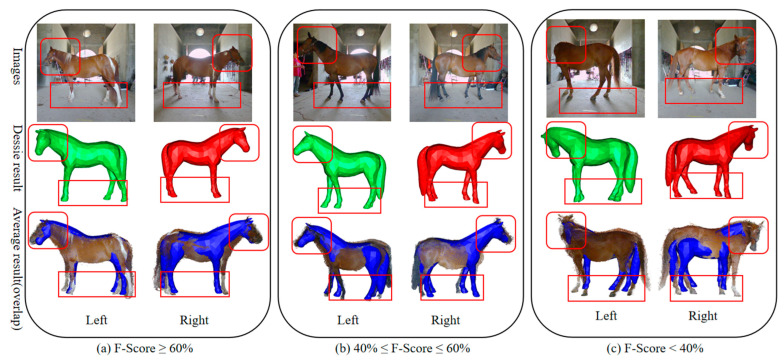
Visualisation of DESSIE single-view meshes and multi-view averaged mesh–reference point cloud overlap under three F-Score quality groups. Each group is organised into three rows: raw multi-view RGB images (top), left- and right-view DESSIE reconstruction meshes [33] (green and red, middle), and averaged multi-view meshes overlapped with reference RGB-D point clouds (bottom). Blue regions indicate overlapping areas. (**a**) High-quality samples (F-Score ≥ 60%) show relatively symmetric postures, complete torso and limb geometry, and close overall overlap between the averaged mesh and the reference point cloud. (**b**) Medium-quality samples (40% ≤ F-Score < 60%) show generally upright postures and well-aligned torso regions, while mild posture asymmetry leads to blurred limb surfaces and incomplete local overlap. (**c**) Low-quality samples (F-Score < 40%) show severely skewed or twisted standing postures, resulting in distorted averaged meshes and limited overlap with the reference point cloud. Red boxes highlight areas exhibiting obvious reconstruction discrepancies.

According to the F-Score calculated via Equation (24), the original dataset contains 34 captured horse samples. However, severe inherent defects exist in the raw multi-view point clouds of partial samples from Daoxianghu, leading to extremely abnormal outlier evaluation metrics that deviate drastically from realistic reconstruction performance. To eliminate the statistical bias caused by these invalid outliers and guarantee the reliability of group-averaged quantitative results, these defective samples are excluded from all statistical calculations. The remaining 32 valid samples are split into three subgroups with thresholds of 60% and 40%, and the averaged Chamfer Distance and F-Score metrics for each stratified group are summarised in Table 7. Skewed standing postures mainly induce two typical reconstruction errors: insufficient fine geometric recovery of slender leg structures in the medium-F subgroup and severe global whole-body silhouette distortion in the low-F subgroup, as visualised in Figure 13.

Samples with F-Score ≥ 60% are categorised as the high-quality reconstruction group. These horses stand in relatively symmetric postures, so the left and right views provide consistent body contour information. The multi-view average mesh achieves tight alignment with the ground truth along the X, Y and Z spatial axes. Both trunk and leg contours are completely restored, resulting in low Chamfer Distance and superior comprehensive F-Score. When serving as the input of non-rigid registration, such complete average meshes rarely produce geometric mismatches in limb and torso regions, as shown in Figure 13a.

The medium-F subgroup (40% ≤ F-Score < 60%) contains samples with regular upright postures and well-fitted trunk meshes. However, mild asymmetry or slight turning of the body causes the slender leg structures to appear differently in the left and right views. During multi-view averaging, this inconsistency leads to fuzzy limb surfaces and partially missing local geometric details on the average mesh. These inherent local defects directly impair the overall F-Score. The typical leg blurring and incomplete reconstruction artifacts of this subgroup are illustrated in Figure 13b, which intuitively demonstrates how posture-induced view inconsistency degrades the comprehensive F-Score of registration results.

The low-F subgroup with F-Score < 40% consists of samples with severely skewed, torsional, or asymmetric standing postures. Although complete multi-view point cloud data are collected, the inconsistent body outlines captured from multiple viewing angles lead to distorted average mesh shapes in advance. Such flawed mesh inputs trigger obvious global three-dimensional deviation during non-rigid registration and drastically drag down the final F-Score. This category corresponds to the global alignment failure shown in Figure 13c.

**Figure 14:**
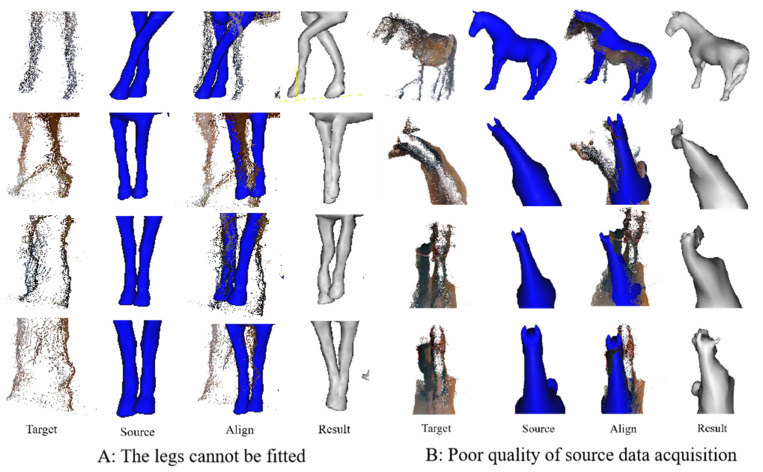
Incorrect fitting results in non-rigid registration. This figure presents two typical incorrect fitting cases in non-rigid registration experiments: Part (**A**) shows leg fitting errors caused by nearest-neighbour matching deviation, while Part (**B**) displays fitting limitations induced by defects in original sequence data acquisition. Target denotes ground-truth (GT) point cloud, Source (blue mesh) refers to the averaged mesh, and Result (grey mesh) is the mesh after non-rigid registration.

We further exhibit two representative incorrect fitting scenarios induced by flawed average mesh inputs in Figure 14 to intuitively interpret the specific alignment failure patterns.

Combining the quantitative statistics in Table 7 with the visual examples in Figure 13 and Figure 14, the results indicate that the geometric quality of the averaged mesh is an important prerequisite for reliable non-rigid registration. Skewed or asymmetric postures may introduce global shape distortion in the averaged mesh, while insufficient reconstruction of slender limb structures can lead to local missing geometry. These defects reduce the geometric agreement between the reconstructed mesh and the reference point cloud and may further affect the accuracy of subsequent non-rigid registration.

## 4. Discussion

Based on the qualitative and quantitative results in Section 3.2, non-rigid registration achieves significant optimisation on both datasets. For the Daoxiang Lake and Zuping datasets, the relative error reduction rates of body height (BH) in the MAE metric reach 63.59% and 67.82%, respectively, with corresponding relative reductions in MAPE exceeding 64% (64.44% for Daoxiang Lake and 68.32% for Zuping). This indicates that the method can effectively correct local geometric deviations induced by image reconstruction (e.g., torso contour deformation and blurred limb details in reconstructed models) and improve the accuracy of anatomical measurements.

Meanwhile, the Chamfer Distance (CD) metric decreases from 11.46 cm (Daoxiang Lake) and 10.86 cm (Zuping) to 5.06 cm and 5.36 cm, respectively, with a relative reduction in geometric fitting error of over 50%. Improvements in Precision, Recall, and F-Score further confirm the method’s effectiveness: For the Daoxiang Lake dataset, Precision increases from 62.63% to 87.70% and Recall from 52.95% to 83.78%; for the Zuping dataset, Precision rises from 66.12% to 88.24% and Recall from 52.33% to 77.87%. Additionally, the F-Score reaches 85.54% (Daoxiang Lake) and 82.70% (Zuping), reflecting that non-rigid registration not only optimises geometric morphology but also enhances the model’s ability to capture key equine features (e.g., joint contours and torso curves), thereby reducing feature loss caused by occlusion or view deviation. This consistency with relative MAE/MAPE reductions in occlusion-vulnerable dimensions (e.g., Cannon Circumference (CC), with 16.58% and 9.94% reductions for Daoxiang Lake and Zuping, respectively) demonstrates the method’s stability in scenarios involving complex morphological features. It should be noted objectively that during the non-rigid registration experiment, two key issues were identified that have a significant impact on the registration effect. Firstly, the nearest-neighbour matching step in the registration phase is prone to deviations, specifically manifested as the matching results of feature points on the legs of the source model (i.e., the average model generated in the early stage) deviating from the corresponding regions of the target model (real point cloud or target reconstruction model). This issue can be intuitively observed in Part A of Figure 14 Secondly, defects in the acquisition of original sequence data restrict the registration accuracy, mainly including two aspects: one is the incomplete data acquisition from some views, leading to the loss of local feature information; the other is the asynchrony in the acquisition time of multi-view data, resulting in slight differences in the target posture. Such defects are clearly reflected in Part B of Figure 14.

Furthermore, the MAE and MAPE for body length (BL) and heart girth (HG) on the Zuping dataset show slight increases. It is speculated that this may be related to differences in posture within local samples of this dataset (such as deviations in the standing angle of horses) or to the uneven density of the original point cloud. However, from the perspective of overall metrics (CD reduction exceeding 50%, significant decreases in MAE/MAPE for core dimensions, and an F-Score increase exceeding 24%), the optimisation benefits of non-rigid registration still dominate. The consistent results from the two datasets indicate that this method can not only improve the quantitative accuracy and geometric fitting degree of 3D horse reconstruction but also exhibit good dataset adaptability. Moreover, the issues identified above provide a clear direction for subsequent method improvements (such as optimising the leg feature-point matching strategy and improving the data acquisition process), laying a more solid foundation for research on horse reconstruction under complex postures.

Although this study has achieved significant progress in livestock 3D reconstruction and body measurement technology, it still has limitations.

1. The reconstruction of the initial parametric model relies on the quality of the input image. When the horse is subject to severe occlusion (such as limb overlap, or occlusion of key morphological regions by environmental debris) or extreme postures (such as lying down and violent twisting), the robustness of the decoupled learning framework in capturing morphological features will decrease, which affects the accuracy of subsequent model reconstruction and parametric calculation.
2. The optimisation of non-rigid fitting first manifests in its high dependence on the initial parametric model. When the initial parametric model fails to achieve “rough alignment”, non-rigid registration can hardly correct posture deviations, and errors will be amplified. Due to dynamic motion, the current bounding box range cannot fully cover the motion trajectory, and partial point cloud data loss is likely to cause registration deviations, resulting in deficiencies in scene adaptability.
3. The current technical solution is designed for horse morphology. Although it has some migration potential within the template framework, when directly applied to other livestock with large body shape differences (such as cattle and sheep), it is necessary to reuse their corresponding high-quality parametric models and adjust the non-rigid fitting threshold. A universal livestock image reconstruction and measurement system has not yet been formed.
4. The proposed method is trained and validated exclusively on images of live horses and relies on equine morphological priors encoded in the hSMAL template. For horse statue images, if the statue has realistic proportions and anatomical structures highly consistent with live horses, the reconstruction pipeline will still generate a 3D mesh, but the body measurement results will have no practical biological meaning. If the statue has stylised deformations (e.g., exaggerated muscles and abnormal limb proportions), the reconstruction accuracy will decrease significantly. Currently, the algorithm does not include a preprocessing module to distinguish live horses from statues; adding such a binary classification module would be a straightforward improvement for practical farm deployment.

In future research, we will centre on monocular reconstruction as the core, focusing on enhancing its robustness and practicality for 3D reconstruction and livestock body measurement. To address the limitations mentioned above, we will first optimise reliance on image quality in monocular vision, improve feature capture performance in scenarios with severe occlusion and extreme postures, and provide more stable support for the reconstruction of initial parametric models. Second, we will optimise the monocular non-rigid fitting framework, reduce reliance on the “rough alignment” of the initial model, and adapt to the boundary-constrained requirements of dynamic movements. Finally, based on the existing template framework, we will explore cross-species adaptation schemes to gradually develop a universal monocular 3D reconstruction and body measurement system for livestock.

## 5. Conclusions

This research study focuses on template-based livestock 3D reconstruction and body dimension measurement technology, effectively addressing the key technical issues in this field. A decoupled learning framework is adopted, and relying on hSMAL, this study integrates horse morphological priors and image features to accurately capture the key morphological information of horses, thereby alleviating depth ambiguity and achieving high-precision restoration of 3D structures. For the reconstruction results, coarse-to-fine registration is applied to optimise local geometric distortions in the abdomen and legs of the initial reconstruction model, thereby improving its geometric accuracy. Meanwhile, an automated body dimension measurement method that combines spatial distance calculation and surface perimeter fitting is proposed to realise automatic body measurement, and data accuracy is verified through qualitative and quantitative evaluations.

As shown in the experimental results, the horse mesh optimised by non-rigid fitting shows a significant performance improvement compared with the initial average mesh. These results indicate that non-rigid registration can effectively improve the geometric fitting of the horse mesh and enhance the reconstruction of fine anatomical details.

In conclusion, this study optimises the technical process of image-to-3D reconstruction for livestock, provides a feasible solution for the automatic measurement of livestock body dimensions in large-scale breeding, and also offers technical references for the application and promotion of image-to-3D reconstruction in the field of precision livestock farming.

## Figures and Tables

**Table 1:** Key point mapping.

Key Point ID ^1^	Body Part Name
0	Mouth
1 (18)	Right (Left) eye
2 (19)	Right (Left) ear
3 (20)	Right (Left) front_neck
4 (21)	Right (Left) front_paw
5 (22)	Right (Left) front_knee
6 (23)	Right (Left) front_leg
7 (24)	Right (Left) back_paw
8 (25)	Right (Left) back_knee
9 (26)	Right (Left) back_leg
10 (27)	Right (Left) Elbow
11 (28)	Right (Left) Mid
12 (29)	Right (Left) P
13	Tail_top
14 (30)	Right (Left) front_neck2
15 (31)	Right (Left) front_paw2
16 (32)	Right (Left) back_paw2
17 (33)	Right (Left) back_knee2

^1^ For bilaterally paired landmarks, the ID outside the parentheses corresponds to the right-side body part, while the ID in parentheses corresponds to the left-side body part. For example, “1 (18)” represents the right eye (ID 1) and left eye (ID 18), respectively.

**Table 2:** Quantitative comparison of the averaged meshes and the non-rigid registration results on the Daoxiang Lake (d) and Zuping (z) datasets (“↓” indicates that a lower value represents better performance; the upward arrow “↑” indicates that a higher value represents better performance.).

Dataset/Metric	CD ^3^ ↓ (cm)	Precision ^4^ ↑ (%)	Recall ^4^ ↑ (%)	F-Score ^4^ ↑ (%)
Average ^1^ (d)	11.46	62.63	52.95	57.14
Non-rigid ^2^ (d)	**5.06**	**87.70**	**83.78**	**85.54**
Average (z)	10.86	66.12	52.33	58.33
Non-rigid (z)	**5.36**	**88.24**	**77.87**	**82.70**

^1^ “Average” denotes the fused mesh obtained by averaging the aligned left- and right-view reconstruction results before non-rigid registration, whereas ^2^ “Non-rigid” denotes the mesh obtained after non-rigid registration. ^3^ CD represents Chamfer Distance (cm), for which lower values indicate better performance. ^4^ Precision, Recall, and F-Score are reported as percentages (%), with higher values indicating better performance. Bold values represent results with lower errors in comparative analysis.

**Table 3:** Comparison of manual reference and model-derived body measurements for horses in the Daoxiang Lake (d) dataset. (GT: ground truth; Average: average template results; Non-rigid: fitting results) (- indicates no value).

	BH ^4^ (cm)			BL ^5^ (cm)			HG ^6^ (cm)			CC ^7^ (cm)		
	GT ^1^	Average ^2^	Non-Rigid ^3^	GT	Average	Non-Rigid	GT	Average	Non-Rigid	GT	Average	Non-Rigid
d1	167	162	164.25	162	153	167.77	193	197.85	194.72	21.00	32.17	30.94
d2	161	142	-	154	134	-	189	182.30	-	20.00	28.61	-
d3	166	157	-	167	157	-	198	200.48	-	21.00	32.55	-
d4	172	170	172.68	174	166	172.09	204	213.37	198.92	22.00	35.87	28.49
d5	171	167	169.62	170	156	166.65	189	204.42	190.76	22.00	33.42	33.36
d6	167	125	-	166	132	148.01	204	167.11	200.61	22.00	27.01	26.40
d7	167	165	170.59	170	158	170.00	193	205.60	197.89	20.00	32.64	30.45
d8	166	156	164.8	163	153	159.76	189	195.12	189.53	21.00	31.33	28.94
d9	164	-	-	156	-	-	188	-	-	19.50	-	-
d10	165	161	163.94	164	154	163.01	201	199.49	199.07	20.50	32.20	-
d11	156	145	155.55	159	146	149.62	192	183.47	194.04	19.50	30.41	33.32
d12	-	165	-	-	154	-	191	200.86	192.96	20.00	31.8	28.22
d13	165	155	-	160	149	-	194	191.81	-	20.00	30.48	-
d14	167	-	-	168	-	-	208	-	-	21.50	-	-
d15	143	138	143.00	141	136	148.87	178	174.11	181.14	19.00	28.01	27.90
d16	161	163	160.63	160	158	171.84	193	201.89	203.14	23.00	32.06	29.76
d17	140	133	-	143	129	-	171	165.00	-	22.00	26.10	-
d18	146	142	144.44	158	135	147.46	176	175.45	176.50	23.00	28.18	23.85

^1^ GT denotes true measurement values. ^2^ Average denotes the body measurements obtained from the averaged mesh generated by fusing the aligned left- and right-view reconstruction results before non-rigid registration, ^3^ whereas Non-rigid denotes the measurements obtained from the mesh after non-rigid registration. ^4^ BH, ^5^ BL, ^6^ HG, and ^7^ CC represent body height, body length, heart girth, and cannon circumference, respectively. All measurements are reported in centimetres (cm). “-” indicates that the corresponding measurement was unavailable.

**Table 4:** Comparison of manual reference and model-derived body measurements for horses in the Zuping (z) dataset. (GT: ground truth; Average: average template results; Non-rigid: fitting results).

		BH ^4^ (cm)			BL ^5^ (cm)			HG ^6^ (cm)			CC ^7^ (cm)	
	GT ^1^	Average ^2^	Non-Rigid ^3^	GT	Average	Non-Rigid	GT	Average	Non-Rigid	GT	Average	Non-Rigid
z1	171.00	173	172.28	165.00	161	179.05	207	211.89	207.63	23.00	33.69	31.14
z2	166.00	168	167.47	170.00	164	181.12	194	208.43	214.01	21.00	32.90	31.15
z3	170.00	158	168.69	163.00	152	163.71	193	194.35	201.66	21.00	31.22	31.54
z4	170.00	163	170.35	164.00	158	164.29	193	204.29	207.42	21.00	32.68	31.95
z5	168.00	168	168.10	169.00	159	175.53	193	205.59	207.05	22.00	32.75	32.64
z6	169.00	141	158.96	167.00	139	151.19	190	197.63	201.50	25.00	28.57	28.73
z7	175.00	144	160.99	173.00	143	159.20	197	182.64	186.62	22.00	29.19	29.23
z8	164.00	158	164.64	162.00	155	175.32	189	196.67	201.52	21.00	31.37	30.51
z9	169.00	140	155.33	169.00	137	154.38	195	172.65	186.68	21.50	27.43	27.77
z10	161.00	156	162.87	168.00	151	160.58	190	190.90	186.63	20.00	30.43	26.76
z11	162.00	175	165.26	162.00	168	185.42	191	215.01	207.20	19.50	33.75	31.50
z12	153.00	163	157.52	154.00	159	175.69	183	202.32	202.16	19.00	32.05	31.00
z13	159.00	165	158.89	157.00	156	166.98	181	201.03	201.45	19.00	31.66	31.29
z14	150.00	149	149.77	153.00	147	174.08	176	187.87	194.14	19.00	30.05	27.64
z15	165.50	161	166.42	165.50	155	171.62	197	199.96	208.83	20.00	31.85	29.60
z16	145.50	160	146.70	153.00	154	171.91	178	197.81	194.82	20.00	31.73	32.22

^1^ GT denotes true measurement values. ^2^ Average denotes the body measurements obtained from the averaged mesh generated by fusing the aligned left- and right-view reconstruction results before non-rigid registration, ^3^ whereas Non-rigid denotes the measurements obtained from the mesh after non-rigid registration. ^4^ BH, ^5^ BL, ^6^ HG, and ^7^ CC represent body height, body length, heart girth, and cannon circumference, respectively.

**Table 5:** Table of MAE and MAPE for the Daoxiang Lake dataset.

Metric	Dataset	BH	BL	HG	CC
MAE (cm)	Average	9.20	13.06	8.49	9.71
	Non-rigid	**3.35 ^1^**	**6.63**	**3.09**	**8.10**
MAPE (%)	Average	5.68	8.10	4.38	46.44
	Non-rigid	**2.02**	**4.15**	**1.59**	**39.13**

^1^ Bold numbers denote the optimal performance in comparative results.

**Table 6:** Table of MAE and MAPE for the Zuping dataset.

Metric	Dataset	BH	BL	HG	CC
MAE (cm)	Average	10.69	**11.28**	**12.22**	10.46
	Non-rigid	**3.44 ^1^**	12.43	12.90	**9.42**
MAPE (%)	Average	6.47	**6.77**	**6.48**	51.05
	Non-rigid	**2.05**	7.69	6.87	**45.90**

^1^ Bold numbers denote the optimal performance in comparative results.

**Table 7:** Average 3D evaluation metrics under different reconstruction quality groups stratified by the F-Score of the averaged mesh. (“↓” indicates that a lower value represents better performance; the upward arrow “↑” indicates that a higher value represents better performance.).

Sample Subgroup Stratified by F-Score	Number of Samples	CD ↓ (cm)	F-Score ↑ (%)
High-quality group (F-Score ≥ 60%)	8	8.99	65.96
Medium-quality group (40% ≤ F-Score < 60%)	23	11.50	55.61
Low-quality group (F-Score < 40%)	1	20.08	35.93

## Data Availability

The datasets analysed in this study were provided by the authors of the original study [37] upon request and with permission. As the authors of the present study are not permitted to redistribute the data, requests for data access should be directed to the corresponding author of the original study.

## Supplementary Materials

The following supporting information can be downloaded at: https://www.mdpi.com/article/10.3390/ani16152397/s1, Video S1: Pointcloud process.mp4; Video S2: Non-rigid registration process.mp4; Video S3: Automated Body measurement.mp4; Video S4: 3D Evaluation Metrics.mp4.

## Abbreviations

The following abbreviations are used in this manuscript:

PLF	Precision Livestock Farming
CD	Chamfer Distance
SMPL	Skinned Multi-Person Linear Model
SMAL	Skinned Multi-Animal Linear Model
GCN	Graph Convolutional Network
hSMAL	Horse-specific version of SMAL
RANSAC	Random Sample Consensus
ICP	Iterative Closest Point
ARAP	As-Rigid-As-Possible
BH	Body Height
BL	Body Length
HG	Heart Girth
CC	Cannon Circumference
PCA	Principal Component Analysis
MAE	Mean Absolute Error
MAPE	Mean Absolute Percentage Error
